# Supplementary material for: Spiritual Connectivity Intervention for Individuals with Depressive Symptoms: A Randomized Control Trial
Source: Healthcare (Basel). 2024 Aug 12;12(16):1604. doi: 10.3390/healthcare12161604 (PMC11354055; doi:10.3390/healthcare12161604)
Supplement: Supplementary file 1 [file healthcare-12-01604-s001.zip › Supplementary Figures and Tables.pdf]

Supplementary Materials for Spiritual Connectivity Intervention for Individuals with Depressive Symptoms: A Randomized Control Trial

Table S1. Concept mapping on integration of treatment strategies

Session	Content	Integration with CBT, positive psychology, and spirituality/religiosity
1	Spirituality, mental health, and depression	-Introducing cognitive reframing techniques to explore personal meaning of spirituality.<br>-Implementing positive strategies to introduce the concept of mental health and depression.<br>-Incorporating spiritual and religious beliefs to provide a foundation for understanding mental health and spirituality.
2	Connectedness	- Utilizing CBT techniques to challenge and reframe thoughts about connection with oneself and others.<br>-Applying positive psychology strategies to enhance feelings of connectedness and belonging.<br>-Incorporating spiritual practices and group rituals to strengthen connections and promote spiritual connectedness.
3	Forgiveness and freedom	-Implementing CBT exercises to address cognitive distortions related to forgiveness and freedom, while addressing individual uniqueness.<br>-Incorporating positive psychology interventions to explore steps towards forgiveness and promote emotional liberation.<br>-Integrating spiritual teachings on forgiveness and freedom to provide a framework for healing and reconciliation, and stronger interpersonal connection.
4	Suffering and transcendence	- Using CBT to reframe beliefs about suffering and develop coping strategies.<br>-Integrating positive psychology strategies to promote resilience, courage, and strength during times of suffering.<br>-Incorporating spiritual perspectives on suffering and transcendence to find deeper meaning and purpose in difficult experiences. Encourage shared experiences and discussions that promote mutual support and spiritual growth.
5	Hope	-CBT technique to challenge pessimistic thoughts and promoting optimistic.<br>-Integrating positive psychology interventions to foster a sense of optimism and hope.<br>-Incorporating spiritual teachings on hope and faith to inspire resilience and perseverance in challenging times. Foster a supportive environment that nurtures hope and encourages interpersonal connections.
6	Gratitude	- Incorporating CBT to address cognitive biases and enhance gratitude practice.<br>-Applying positive psychology principles to explore personal ways of expressing gratitude and enhance well-being.<br>- Incorporating spiritual practices of gratitude and thankfulness to cultivate a sense of spiritual well-being and connection. Sharing gratitude practices within the group to strength bonds and promote a sense of community.
7	Relapse prevention and spiritual growth	- Implementing CBT strategies to identify triggers and develop relapse prevention plans.<br>-Incorporating positive psychology strategies to develop coping mechanisms and promote spiritual growth in times of challenge<br>- Integrating spiritual practices and teachings for relapse prevention and spiritual resilience in the face of difficulties. Establishing ongoing support networks and spiritual practices to sustain growth and connection beyond the sessions.
8	Wrap up and celebration	-Implementing positive psychology interventions to celebrate participants' accomplishments and foster a sense of achievement.<br>- Integrating CBT to allow participants to reflect on their learning and emotional growth throughout the sessions.<br>- Incorporating spiritual rituals and communal celebrations to acknowledge progress, growth, and spiritual connections within the group. Cultivating a sense of community and shared achievement through group reflections and celebratory activities.

Note: CBT: cognitive behavior therapy

Table S2. Content outline of the spiritual connectivity intervention.

Session	Content	Goals
1	Spirituality, mental health, and depression	-To explore personal meaning of spirituality<br>-To introduce the concept of mental health and depression
2	Connectedness	-To enhance the experience of connecting with oneself, others, environment and larger meaning<br>- Foster a supportive environment that nurtures hope and encourages interpersonal connections
3	Forgiveness and freedom	-To review forgiveness and freedom<br>-To explore steps towards forgiveness while fostering interpersonal connections
4	Suffering and transcendence	-To explore meaning in suffering<br>-To explore strategies to cultivate courage and strength during challenging times, emphasizing communal support
5	Hope	-To encourage participants to discuss meaning of hope<br>-To discuss strategies to maintain hope and build connections within the group
6	Gratitude	-To explore personal expressions of gratitude within a supportive community setting
7	Relapse prevention and spiritual growth	-To explore participants' early signs of relapse and identify sources of stressors<br>-To explore coping strategies and foster spiritual growth within interpersonal connections
8	Wrap up and celebration	-To celebrate participants' accomplishment<br>-To encourage reflections on learning and emotional experiences from previous sessions, highlighting the social and spiritual connections formed

Weekly assignment tasks, daily meditation & prayer are included in the Intervention

Table S3. Checklist for self-monitoring of the treatment sessions.

Skills Competence of Interventionist	Always/ Almost Always	Sometimes	Missed Opportunity
1. Actively engages all participants in the discussion.			
2. Actively listens when a participant is talking.			
3. Communicates with all participants in a respectful, positive, and non-judgmental manner.			
4. Appropriately reinforces participants' ideas and opinions.			
5. Correctly conveys/communicates the program's principles.			
6. Communicates to participants that the participants are experts about their own problems.			
8. Facilitates sharing of ideas among the participants.			
9. Does not impose own ideas on the participants.			
10. Effectively responds when the participants are resistant to new strategies or ideas.			
11. Effectively manages challenging behavior from the participants in the group (e.g., monopolizing, anger, prolonged silence).			
12. Maintains a good pace for group discussions (not too fast, not too slow).			
13. Effectively uses role-play or group activities to teach a principle or strategy.			
14. Builds on the participants' knowledge by incorporating the strategies discussed in previous sessions into this session.			

1 = skill rarely or never demonstrated (skill demonstrated < 25% of the time);

2 = skill sometimes/occasionally demonstrated (skill demonstrated 25–75% of the time);

3 = skill consistently demonstrated (skill demonstrated > 75% of the time); Modified from the Fidelity Checklist [1].

Table S4. Characteristics and reliability coefficients of secondary outcome measures.

Measure	# of Items	Response Options	Scoring Range	Reliability (Cronbach's α)
Daily Spirituality Experience Scale (DSES)	16	1 ("Never or almost never") to 6 ("Many times"); 1 ("Not close") to 4 ("As close as possible")	16 to 94	T0: 0.942, T1: 0.958, T2: 0.971
State Hope Scale (SHS)	6	1 ("Definitely false") to 8 ("Definitely true")	6 to 48	T0: 0.864, T1: 0.904, T2: 0.886
Agency Thinking (SHS-Agency)	3	1 ("Definitely false") to 8 ("Definitely true")	3 to 24	T0: 0.915, T1: 0.927, T2: 0.902
Pathway Thinking (SHS-Pathway)	3	1 ("Definitely false") to 8 ("Definitely true")	3 to 24	T0: 0.443, T1: 0.710, T2: 0.651
Meaning in Life Questionnaire (MLQ)	10	1 ("Absolutely untrue") to 7 ("Absolutely true")	5 to 35	T0: 0.749, T1: 0.816, T2: 0.824
Presence of Meaning in Life (MLQ-Presence)	10	1 ("Absolutely untrue") to 7 ("Absolutely true")	5 to 35	T0: 0.782, T1: 0.896, T2: 0.883
Search of Meaning in Life (MLQ-Search)	10	1 ("Absolutely untrue") to 7 ("Absolutely true")	5 to 35	T0: 0.482, T1: 0.515, T2: 0.548
Rosenberg Self-Esteem Scale (RSES)	10	1 (“Strongly disagree”) to 4 (“strongly agree”)	10 to 40	T0: 0.824, T1: 0.793, T2: 0.850
Multidimensional Scale of Perceived Social Support (MSPSS)	12	1 (very strongly disagree”) to 7 (“very strongly agree”)	12 to 84	T0: 0.931, T1: 0.926, T2: 0.943
Perceived support from family (MSPSS-family)	4	1 (very strongly disagree”) to 7 (“very strongly agree”)	4 to 28	T0: 0.882, T1: 0.901, T2: 0.933
Perceived support from friends (MSPSS-friend)	4	1 (very strongly disagree”) to 7 (“very strongly agree”)	4 to 28	T0: 0.891, T1: 0.912, T2: 0.921
Perceived support from significant others (MSPSS-Sig.)	4	1 (very strongly disagree”) to 7 (“very strongly agree”)	4 to 28	T0: 0.885, T1: 0.886, T2: 0.887

Table S5. Diagnosis of participants.

Faith-based Intervention group			Waitlist control group		
Diagnosis	No. of participants	%	Diagnosis	No. of participants	%
Depression	3	10.71	Depression	7	24.14
Anxiety	1	3.57	Anxiety	--	--
Anxiety Depression	3	10.71	Anxiety Depression	3	10.34
Bipolar disorder	--	---	Bipolar disorder	2	6.90
Postnatal depression	1	3.57	Postnatal depression	--	--
Adjustment disorder	--	--	Adjustment disorder	1	3.45
Nil	20	71.44	Nil	16	55.17
Total	28	100	Total	29	100

Table S6. Number of sessions attended by participants.

Faith-based Intervention group (T0-T1)			Waitlist control group (T1-T2)		
No. of sessions attended	No. of participants	%	No. of sessions attended	No. of participants	%
0	2	7.14	0	--	--
1	--	--	1	--	--
2	2	7.14	2	1 (quitted)	3.45
3	--	--	3	1	3.45
4	2	7.14	4	--	--
5	--	--	5	2	6.90
6	4	14.29	6	3	10.34
7	3	10.71	7	5	17.24
8	15	53.58	8	17	58.62
Total	28	100	Total	29	100

Table S7. The within-group difference in depression, anxiety, spiritual experience, hope, meaning in life, self-esteem, and perceived social support at T0, T1 and T2 in the intervention group and the waitlist control group: intention-to-treat analysis.

Measures	Intervention (n = 28) Mean ± SD	Cohen's d [95% CI]	Waitlist control (n = 29) Mean ± SD	Cohen's d [95% CI]
**Primary outcomes**				
Depression (PHQ-9)				
T0	9.07 ± 4.62###		9.93 ± 3.92 ###	
T1 (T1 vs T0)	2.93 ± 3.93	-1.452 [-0.896, -2.007] ***	10.14 ± 4.85	0.053 [-0.416, 0.525]
T2 (T2 vs T0)	3.46 ± 4.12	-1.325 [-0.808, -1.842] ***	2.97 ± 2.53	-1.794 [-1.042, -2.546] ***
Anxiety (GAD-7)				
T0	8.21 ± 4.90###		8.90 ± 4.56 ###	
T1 (T1 vs T0)	3.14 ± 4.26	-1.124 [-0.630, -1.618] ***	8.24 ± 4.02	-0.172 [-0.628, 0.284]
T2 (T2 vs T0)	3.50 ± 4.35	-1.045 [-0.572, -1.518] ***	2.83 ± 4.35	-1.595 [-0.901, -2.289] ***
**Secondary outcomes**				
Spiritual experience (DSES)				
T0	57.39 ± 12.44#		55.66 ± 13.19###	
T1 (T1 vs T0)	61.54 ± 12.38	0.308 [0.070, 0.546] **	54.83 ± 13.83	-0.059 [-0.345, 0.226]
T2 (T2 vs T0)	61.79 ± 15.29	0.327 [-0.062, 0.716]	62.21 ± 14.94	0.468 [0.138, 0.797] ***
Hope (SHS)				
T0	27.82 ± 8.24##		23.14 ± 8.72###	
T1 (T1 vs T0)	32.79 ± 6.47	0.633 [0.139, 1.127] **	22.69 ± 8.29	-0.054 [-0.481, 0.372]
T2 (T2 vs T0)	32.43 ± 8.63	0.588 [0.011, 1.165] *	29.69 ± 7.63	0.796 [0.182, 1.411] **
Agency thinking (SHS-Agency)				
T0	12.61 ± 5.05###		10.17 ± 5.60###	
T1 (T1 vs T0)	15.82 ± 3.81	0.702 [0.185, 1.220] **	9.76 ± 4.80	-0.083 [-0.475, 0.309]
T2 (T2 vs T0)	15.79 ± 4.78	0.694 [0.081, 1.307] *	14.28 ± 4.50	0.822 [0.250, 1.394] ***
Pathway thinking (SHS-Pathway)				
T0	15.21 ± 3.55#		12.97 ± 3.64##	
T1 (T1 vs T0)	16.96 ± 3.12	0.479 [0.012, 0.946] *	12.93 ± 4.15	-0.009 [-0.500, 0.482]
T2 (T2 vs T0)	16.64 ± 4.21	0.391 [-0.140, 0.922]	15.41 ± 3.66	0.640 [0.047, 1.233] *
Meaning in life (MLQ)				

T0	46.89 ± 10.24		41.62 ± 9.73#	
T1 (T1 vs T0)	49.25 ± 9.79	0.232 [-0.176, 0.641]	39.93 ± 12.88	-0.151 [-0.736, 0.434]
T2 (T2 vs T0)	49.29 ± 10.38	0.236 [-0.183, 0.656]	48.31 ± 10.75	0.597 [0.083, 1.112] **
Presence of meaning in life (MLQ-Presence)				
T0	20.57 ± 6.64		18.35 ± 6.85##	
T1 (T1 vs T0)	22.71 ± 6.00	0.338 [-0.076, 0.751]	17.03 ± 8.75	-0.178 [-0.659, 0.302]
T2 (T2 vs T0)	23.39 ± 6.40	0.444 [-0.027, 0.916]	22.21 ± 6.23	0.525 [0.103, 0.948] **
Search of meaning in life (MLQ-Search)				
T0	26.32 ± 4.64		23.28 ± 7.59#	
T1 (T1 vs T0)	26.54 ± 4.60	0.043 [-0.336, 0.421]	22.90 ± 6.96	-0.054 [-0.562, 0.453]
T2 (T2 vs T0)	25.89 ± 5.80	-0.085 [-0.488, 0.318]	26.10 ± 6.34	0.405 [-0.112, 0.922]
Self-esteem (RSES)				
T0	25.21 ± 5.06###		24.55 ± 5.09###	
T1 (T1 vs T0)	27.54± 3.83	0.503 [0.080, 0.926] **	25.21 ± 3.91	0.151 [-0.183, 0.486]
T2 (T2 vs T0)	28.11 ± 4.86	0.627 [0.109, 1.145] **	27.45 ± 3.88	0.669 [0.151, 1.187] **
Perceived social support (MSPSS)				
T0	51.82 ± 14.36##		48.86 ± 13.67#	
T1 (T1 vs T0)	58.68 ± 12.82	0.515 [0.059, 0.971] *	53.34 ± 10.04	0.397 [-0.307, 1.010]
T2 (T2 vs T0)	56.32 ± 12.68	0.338 [-0.138, 0.814]	57.41 ± 9.76	0.757 [-0.054, 1.567]
Perceived support from family (MSPSS-family)				
T0	16.64 ± 5.36		14.21 ± 5.36#	
T1 (T1 vs T0)	18.32 ± 4.94	0.324 [-0.121, 0.770]	15.76 ± 5.39	0.308 [-0.290, 0.905]
T2 (T2 vs T0)	17.71 ± 5.18	0.207 [-0.261, 0.676]	17.52 ± 4.30	0.657 [0.020, 1.293] *
Perceived support from friends (MSPSS-friend)				
T0	17.14 ± 5.67##		17.03 ± 4.71	
T1 (T1 vs T0)	19.96 ± 5.21	0.540 [0.134, 0.946] **	18.38 ± 3.69	0.330 [-0.412, 1.072]
T2 (T2 vs T0)	18.96 ± 4.75	0.349 [-0.192, 0.890]	19.55 ± 4.75	0.618 [-0.174, 1.409]
Perceived support from significant others (MSPSS-Sig.)				
T0	18.04 ± 5.22#		17.62 ± 5.57#	
T1 (T1 vs T0)	20.39 ± 4.55	0.499 [-0.040, 1.038]	19.21 ± 3.04	0.388 [-0.284, 1.061]
T2 (T2 vs T0)	19.64 ± 4.36	0.340 [-0.084, 0.764]	20.34 ± 3.13	0.667 [-0.135, 1.468]

Note. T0 = baseline, T1 = post intervention for intervention group, baseline for waitlist control group, T2 = 3-month follow-up. Difference among three time points: $^{\#}$ $p < .05$, $^{\#\#}$ $p < .01$, $^{\#\#\#}$ $p <. 001$. Difference between two time points: * $p < .05$, ** $p < .01$, *** $p <. 001$. Effect size (Cohen's *d*): small = 0.20, moderate = 0.50, large = 0.80. Repeated measures ANOVAs and post-hoc tests with Bonferroni corrections were used to compare the parametric data among three and two time points respectively.

Table S8. Marginal means of the outcome variables by the intervention group (SCG) age subgroups at post-intervention (T1).

Outcome variables	Marginal mean (95% CI) of younger subgroup	Marginal mean (95% CI) of older subgroup
Primary outcomes		
Depression (PHQ-9)	6.490 (3.717, 9.263)	1.743 (-0.693, 4.180)
Anxiety (GAD-7)	6.089 (3.255, 8.923)	1.641 (-0.849, 4.132)
Secondary outcomes		
Spiritual experience (DSES)	47.267 (39.987, 54.546)	55.479 (49.083, 61.876)
Hope (SHS)	28.189 (23.166, 33.212)	36.858 (32.444, 41.272)
Agency Thinking (SHS-Agency)	12.957 (10.023, 15.891)	17.869 (15.291, 20.447)
Pathway Thinking (SHS-Pathway)	15.232 (12.714, 17.749)	18.989 (16.777, 21.201)
Meaning in Life (MLQ)	47.104 (39.118, 55.090)	50.784 (43.767, 57.802)
Presence of Meaning in Life (MLQ-Presence)	20.625 (15.324, 25.926)	24.820 (20.162, 29.478)
Search of Meaning in Life (MLQ-Search)	26.479 (22.285, 30.673)	25.964 (22.279, 29.650)
Self-Esteem (RSES)	25.610 (22.931, 28.288)	27.772 (25.419, 30.126)
Perceived Social Support (MSPSS)	51.604 (44.059, 59.149)	62.739 (56.109, 69.369)
Perceived support from family (MSPSS-family)	16.189 (13.037, 19.341)	20.022 (17.252, 22.792)

Perceived support from friends (MSPSS-friend)	17.156 (13.934, 20.378)	21.241 (18.410, 24.072)
Perceived support from significant others (MSPSS-Sig.)	18.259 (15.628, 20.889)	21.476 (19.165, 23.787)

Figure S1. Percentage of participants with 5 points reduction in PHQ-9 scores.

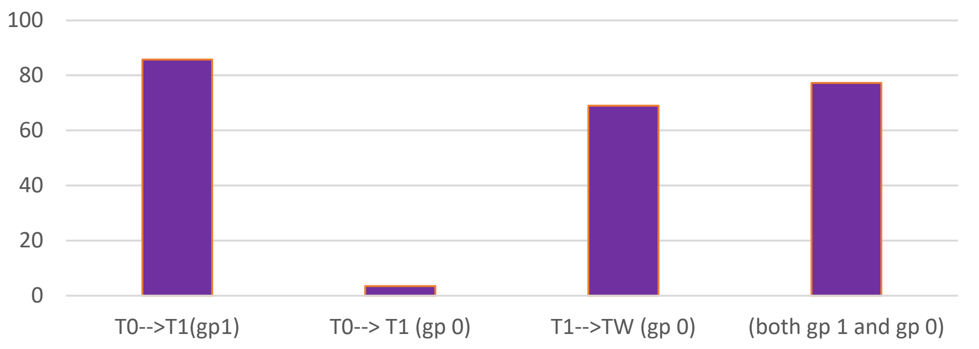

Note: T0=baseline, T1=post intervention for intervention group, baseline for waitlist control group.

TW=post intervention for wait list control group. gp1= intervention group; gp 0=waitlist control group

Table S9. The within-group differences in depression, anxiety, spiritual experience, hope, meaning in life, self-esteem, and perceived social support at T0, T1 and T2 in the intervention group and the waitlist control group: per-protocol analysis.

	**Intervention (n = 26)**		**Waitlist control (n = 28)**	
**Measure**	**Mean ± SD**	**Cohen's d [95% CI]**	**Mean ± SD**	**Cohen's d [95% CI]**
Primary outcomes				
**Depression (PHQ-9)**				
T0	9.00 ± 4.48$^{\#\#\#}$		9.96 ± 3.99$^{\#\#\#}$	
T1 (T1 vs T0)	2.38 ± 3.07	-1.782 [-2.481, -1.084] ***	10.36 ± 4.79	0.101 [-0.372, 0.574]
T2 (T2 vs T0)	2.96 ± 3.44	-1.627 [-2.279, -0.974] ***	2.93 ± 2.57	-1.809 [-2.584, -1.034] ***
**Anxiety (GAD-7)**				
T0	8.04 ± 4.44$^{\#\#\#}$		8.89 ± 4.65$^{\#\#\#}$	
T1 (T1 vs T0)	2.58 ± 3.02	-1.509 [-2.247, -0.771] ***	8.32 ± 4.07	-0.148 [-0.486, 0.189]
T2 (T2 vs T0)	2.96 ± 3.23	-1.403 [-2.030, -0.775] ***	2.71 ± 2.51	-1.605 [-2.398, -0.812] ***
Secondary outcomes				
Spiritual experience (DSES)				
T0	56.42 ± 12.30$^{\#}$		55.64 ± 13.43$^{\#\#\#}$	
T1 (T1 vs T0)	60.88 ± 12.55	0.328 [0.075, 0.582] **	54.75 ± 14.08	-0.063 [-0.354, 0.229]
T2 (T2 vs T0)	61.15 ± 15.65	0.348 [-0.068, 0.764]	62.39 ± 15.18	0.474 [0.138, 0.809] ***
**Hope (SHS)**				
T0	27.88 ± 7.86$^{\#\#}$		23.07 ± 8.87$^{\#\#\#}$	
T1 (T1 vs T0)	33.23 ± 5.55	0.734 [0.162, 1.307] **	22.25 ± 8.10	-0.100 [-0.526, 0.327]
T2 (T2 vs T0)	32.85 ± 8.15	0.682 [0.011, 1.352] *	29.50 ± 7.70	0.780 [0.148, 1.413] **
Agency thinking (SHS-Agency)				
T0	12.58 ± 4.97$^{\#\#\#}$		10.11 ± 5.70$^{\#\#\#}$	
T1 (T1 vs T0)	16.04 ± 3.48	0.788 [0.208, 1.368] **	9.50 ± 4.68	-0.121 [-0.516, 0.273]
T2 (T2 vs T0)	16.00 ± 4.60	0.779 [0.091, 1.468] *	14.18 ± 4.56	0.814 [0.225, 1.403] **
Pathway thinking (SHS-Pathway)				
T0	15.31 ± 3.26$^{\#}$		12.96 ± 3.71$^{\#\#}$	
T1 (T1 vs T0)	17.19 ± 2.62	0.567 [0.013, 1.121] *	12.75 ± 4.11	-0.056 [-0.549, 0.437]
T2 (T2 vs T0)	16.85 ± 3.96	0.463 [-0.169, 1.094]	15.32 ± 3.69	0.614 [-0.045, 1.272]
Meaning in life (MLQ)				
T0	46.73 ± 9.97		42.14 ± 9.49$^{\#\#}$	
T1 (T1 vs T0)	49.27 ± 9.48	0.257 [-0.197, 0.712]	39.57 ± 12.97	-0.229 [-0.802, 0.344]
T2 (T2 vs T0)	49.31 ±10.14	0.261 [-0.205, 0.728]	48.25 ± 10.94	0.544 [0.037, 1.051] *
Presence of meaning in life (MLQ-Presence)				
T0	20.38 ± 6.57$^{\#}$		18.57 ± 6.87$^{\#\#\#}$	
T1 (T1 vs T0)	22.69 ± 5.91	0.367 [-0.085, 0.820]	16.86 ± 8.86	-0.231 [-0.706, 0.244]

T2 (T2 vs T0)	23.42 ± 6.35	0.484 [-0.031, 0.999]	22.21 ± 6.34	0.490 [0.068, 0.912] **
Search of meaning in life (MLQ-Search)				
T0	26.35 ± 4.51		23.57 ± 7.55	
T1 (T1 vs T0)	26.58 ± 4.47	0.047 [-0.370, 0.464]	22.71 ± 7.02	-0.122 [-0.616, 0.372]
T2 (T2 vs T0)	25.88 ± 5.78	-0.093 [-0.538, 0.352]	26.04 ± 6.44	0.351 [-0.160, 0.862]
Self-esteem (RSES)				
T0	25.23 ± 5.13###		24.64 ± 5.16###	
T1 (T1 vs T0)	27.73 ± 3.74	0.542 [0.087, 0.998] **	25.25 ± 3.98	0.139 [-0.203, 0.480]
T2 (T2 vs T0)	28.35 ± 4.84	0.676 [0.118, 1.234] **	27.57 ± 3.89	0.668 [0.137, 1.199] **
Perceived social support (MSPSS)				
T0	52.04 ± 13.41##		50.14 ± 12.02#	
T1 (T1 vs T0)	59.42 ± 11.27	0.614 [0.070, 1.158] *	52.32 ± 8.54	0.220 [-0.326, 0.766]
T2 (T2 vs T0)	56.88 ± 11.26	0.403 [-0.167, 0.973]	56.54 ± 8.69	0.647 [-0.094, 1.388]
Perceived support from family (MSPSS-family)				
T0	16.73 ± 5.01		14.57 ± 5.08#	
T1 (T1 vs T0)	18.54 ± 4.50	0.379 [-0.144, 0.903]	15.32 ± 4.94	0.161 [-0.306, 0.628]
T2 (T2 vs T0)	17.88 ± 4.78	0.242 [-0.309, 0.793]	17.14 ± 3.87	0.552 [0.052, 1.052] *
Perceived support from friends (MSPSS-friend)				
T0	17.23 ± 5.43#		17.50 ± 4.06	
T1 (T1 vs T0)	20.27 ± 4.78	0.625 [0.156, 1.095] **	18.04 ± 3.25	0.149 [-0.478, 0.776]
T2 (T2 vs T0)	19.19 ± 4.30	0.404 [-0.226, 1.033]	19.25 ± 3.44	0.486 [-0.232, 1.205]
Perceived support from significant others (MSPSS-Sig.)				
T0	18.08 ± 5.10#		18.07 ± 5.11	
T1 (T1 vs T0)	20.62 ± 4.27	0.563 [-0.047, 1.173]	18.96 ± 2.80	0.236 [-0.329, 0.802]
T2 (T2 vs T0)	19.81 ± 4.09	0.384 [-0.096, 0.864]	20.14 ± 2.99	0.548 [-0.209, 1.306]

Note. T0 = baseline, T1 = post intervention for intervention group, baseline for waitlist control group, T2 = 3-month follow-up.
Difference among three time points: # $p < .05$, ## $p < .01$, ### $p < .001$. Difference between two time points: * $p < .05$, ** $p < .01$, *** $p < .001$. Effect size (Cohen's *d*): small = 0.20, moderate = 0.50, large = 0.80. Repeated measures ANOVAs and post-hoc tests with Bonferroni corrections were used to compare the parametric data among the overall three time points followed by post hoc pairwise comparisons.

Table S10. The between-group differences in changes in depression, anxiety, spiritual experience, hope, meaning in life, self-esteem, and perceived social support at T1 and T2 in the intervention and waitlist control groups: per-protocol analysis.

Measure	Intervention vs Waitlist control<br>Mean difference [95% CI]	Cohen's *d*
**Difference in the changes at T1**		
Primary outcomes		
Depression (PHQ-9)	-6.893 [-8.582, -5.204]	-2.499***
Anxiety (GAD-7)	-5.336 [-6.876, -3.797]	-2.071***
Secondary outcomes		
Spiritual experience (DSES)	6.808 [2.606, 11.009]	0.969**
Hope (SHS)	10.335 [6.889, 13.781]	1.880***
Agency thinking (SHS-Agency)	6.104 [4.115, 8.093]	1.891***
Pathway thinking (SHS-Pathway)	4.233 [2.418, 6.048]	1.483***
Meaning in life (MLQ)	7.668 [1.671, 13.665]	0.788*
Presence of meaning in life (MLQ-Presence)	4.610 [1.012, 8.208]	0.775*
Search of meaning in life (MLQ-Search)	2.622 [-0.139, 5.383]	0.574
Self-esteem (RSES)	1.917 [0.422, 3.413]	0.764*
Perceived social support (MSPSS)	8.271 [3.312, 13.230]	0.991**
Perceived support from family (MSPSS-family)	3.245 [1.056, 5.434]	0.902**
Perceived support from friends (MSPSS-friend)	2.835 [0.697, 4.972]	0.785*
Perceived support from significant others (MSPSS-Sig.)	2.321 [0.451, 4.191]	0.735*

Note. One-way ANCOVA was used to examine the between group differences. Mean differences and effect size (Cohen's *d*) were computed from estimated marginal means.

Reference

1. Breitenstein, S. M.; Fogg, L.; Garvey, C.; Hill, C.; Resnick, B.; Gross, D. Measuring Implementation Fidelity in a Community-Based Parenting Intervention. *Nurs Res.* **2010,** *59*, 158-165.
