# Supplementary material for: Spiritual Connectivity Intervention for Individuals with Depressive Symptoms: A Randomized Control Trial
Source: Healthcare (Basel). 2024 Aug 12;12(16):1604. doi: 10.3390/healthcare12161604 (PMC11354055; doi:10.3390/healthcare12161604)
Supplement: Supplementary file 1 [file healthcare-12-01604-s001.zip › Supplementary File.pdf]

File S1. Code used for experimental implementation-SPSS output syntax

1. **Demographics**

```
CROSSTABS
  /TABLES=Sex Age Religious Education Marital Occupation
Treatment BY Group
  /FORMAT=AVALUE TABLES
  /STATISTICS=CHISQ
  /CELLS=COUNT ROW COLUMN TOTAL
  /COUNT ROUND CELL
```

2. **Baseline comparison**

```
T-TEST GROUPS=Group(0 1)
  /MISSING=ANALYSIS
  /VARIABLES=T0_DSES_Total T0_SHS_Total T0_SHS_Agency
T0_SHS_Pathway T0_MLQ_total T0_MLQ_Presence
    T0_MLQ_Search T0_RSES_Total T0_MSPSS_total T0_MSPSS_fam
T0_MSPSS_frd T0_MSPSS_sig T0_PHQ9_Total
    T0_GAD7_Total
  /CRITERIA=CI(.95).
```

3. **Within-group repeated measures ANOVA (PHQ9)**

```
GLM T0_PHQ9_Total T1_PHQ9_Total T2_PHQ9_Total BY Group
  /WSFACTOR=time 3 Polynomial
  /MEASURE=PHQ9
  /METHOD=SSTYPE(3)
  /PLOT=PROFILE(time*Group) TYPE=LINE ERRORBAR=CI
MEANREFERENCE=NO YAXIS=AUTO
  /EMMEANS=TABLES(OVERALL)
  /EMMEANS=TABLES(time) COMPARE ADJ(BONFERRONI)
  /EMMEANS=TABLES(Group) COMPARE ADJ(BONFERRONI)
  /EMMEANS=TABLES(Group*time)
  /CRITERIA=ALPHA(.05)
  /WSDESIGN=time
  /DESIGN=Group.
```

4. **Within-group repeated measures ANOVA (GAD7)**

```
GLM T0_GAD7_Total T1_GAD7_Total T2_GAD7_Total BY Group
  /WSFACTOR=time 3 Polynomial
  /MEASURE=GAD7
  /METHOD=SSTYPE(3)
  /PLOT=PROFILE(time*Group) TYPE=LINE ERRORBAR=CI
MEANREFERENCE=NO YAXIS=AUTO
  /EMMEANS=TABLES(OVERALL)
  /EMMEANS=TABLES(time) COMPARE ADJ(BONFERRONI)
```

```
  /EMMEANS=TABLES(Group) COMPARE ADJ(BONFERRONI)
  /EMMEANS=TABLES(Group*time)
  /CRITERIA=ALPHA(.05)
  /WSDESIGN=time
  /DESIGN=Group.
```

5. **Within-group repeated measures ANOVA (DSES)**

```
GLM T0_DSES_Total T1_DSES_Total T2_DSES_Total BY Group
  /WSFACTOR=time 3 Polynomial
  /MEASURE=DSES
  /METHOD=SSTYPE(3)
  /PLOT=PROFILE(time*Group) TYPE=LINE ERRORBAR=CI
MEANREFERENCE=NO YAXIS=AUTO
  /EMMEANS=TABLES(OVERALL)
  /EMMEANS=TABLES(time) COMPARE ADJ(BONFERRONI)
  /EMMEANS=TABLES(Group) COMPARE ADJ(BONFERRONI)
  /EMMEANS=TABLES(Group*time)
  /CRITERIA=ALPHA(.05)
  /WSDESIGN=time
  /DESIGN=Group.
```

6. **Within-group repeated measures ANOVA (SHS)**

```
GLM T0_SHS_Total T1_SHS_Total T2_SHS_Total BY Group
  /WSFACTOR=time 3 Polynomial
  /MEASURE=SHS
  /METHOD=SSTYPE(3)
  /PLOT=PROFILE(time*Group) TYPE=LINE ERRORBAR=CI
MEANREFERENCE=NO YAXIS=AUTO
  /EMMEANS=TABLES(OVERALL)
  /EMMEANS=TABLES(time) COMPARE ADJ(BONFERRONI)
  /EMMEANS=TABLES(Group) COMPARE ADJ(BONFERRONI)
  /EMMEANS=TABLES(Group*time)
  /CRITERIA=ALPHA(.05)
  /WSDESIGN=time
  /DESIGN=Group.
```

7. **Within-group repeated measures ANOVA (SHS-agency, pathway)**

```
GLM T0_SHS_Agency T1_SHS_Agency T2_SHS_Agency T0_SHS_Pathway
T1_SHS_Pathway T2_SHS_Pathway BY Group
  /WSFACTOR=time 3 Polynomial
  /MEASURE=SHS_agency SHS_pathway
  /METHOD=SSTYPE(3)
  /PLOT=PROFILE(time*Group) TYPE=LINE ERRORBAR=CI
MEANREFERENCE=NO YAXIS=AUTO
  /EMMEANS=TABLES(OVERALL)
  /EMMEANS=TABLES(time) COMPARE ADJ(BONFERRONI)
  /EMMEANS=TABLES(Group) COMPARE ADJ(BONFERRONI)
  /EMMEANS=TABLES(Group*time)
  /CRITERIA=ALPHA(.05)
```

```
  /WSDESIGN=time
  /DESIGN=Group.
```

8. **Within-group repeated measures ANOVA (MLQ)**

```
GLM T0_MLQ_total T1_MLQ_Total T2_MLQ_Total BY Group
  /WSFACTOR=time 3 Polynomial
  /MEASURE=MLQ
  /METHOD=SSTYPE(3)
  /PLOT=PROFILE(time*Group) TYPE=LINE ERRORBAR=CI
MEANREFERENCE=NO YAXIS=AUTO
  /EMMEANS=TABLES(OVERALL)
  /EMMEANS=TABLES(time) COMPARE ADJ(BONFERRONI)
  /EMMEANS=TABLES(Group) COMPARE ADJ(BONFERRONI)
  /EMMEANS=TABLES(Group*time)
  /CRITERIA=ALPHA(.05)
  /WSDESIGN=time
  /DESIGN=Group.
```

9. **Within-group repeated measures ANOVA (MLQ-presence, search)**

```
GLM T0_MLQ_Presence T1_MLQ_Presence T2_MLQ_Presence
T0_MLQ_Search T1_MLQ_Search T2_MLQ_Search BY
    Group
  /WSFACTOR=time 3 Polynomial
  /MEASURE=MLQ_presence MLQ_search
  /METHOD=SSTYPE(3)
  /PLOT=PROFILE(time*Group) TYPE=LINE ERRORBAR=CI
MEANREFERENCE=NO YAXIS=AUTO
  /EMMEANS=TABLES(OVERALL)
  /EMMEANS=TABLES(time) COMPARE ADJ(BONFERRONI)
  /EMMEANS=TABLES(Group) COMPARE ADJ(BONFERRONI)
  /EMMEANS=TABLES(Group*time)
  /CRITERIA=ALPHA(.05)
  /WSDESIGN=time
  /DESIGN=Group.
```

10. **Within-group repeated measures ANOVA (RSES)**

```
GLM T0_RSES_Total T1_RSES_Total T2_RSES_Total BY Group
  /WSFACTOR=time 3 Polynomial
  /MEASURE=RSES
  /METHOD=SSTYPE(3)
  /PLOT=PROFILE(time*Group) TYPE=LINE ERRORBAR=CI
MEANREFERENCE=NO YAXIS=AUTO
  /EMMEANS=TABLES(OVERALL)
  /EMMEANS=TABLES(time) COMPARE ADJ(BONFERRONI)
  /EMMEANS=TABLES(Group) COMPARE ADJ(BONFERRONI)
  /EMMEANS=TABLES(Group*time)
  /CRITERIA=ALPHA(.05)
  /WSDESIGN=time
  /DESIGN=Group.
```

11. Within-group repeated measures ANOVA (MSPSS)

```
GLM T0_MSPSS_total T1_MSPSS_Total T2_MSPSS_Total BY Group
  /WSFACTOR=time 3 Polynomial
  /MEASURE=MSPSS
  /METHOD=SSTYPE(3)
  /PLOT=PROFILE(time*Group) TYPE=LINE ERRORBAR=CI
MEANREFERENCE=NO YAXIS=AUTO
  /EMMEANS=TABLES(OVERALL)
  /EMMEANS=TABLES(time) COMPARE ADJ(BONFERRONI)
  /EMMEANS=TABLES(Group) COMPARE ADJ(BONFERRONI)
  /EMMEANS=TABLES(Group*time)
  /CRITERIA=ALPHA(.05)
  /WSDESIGN=time
  /DESIGN=Group.
```

12. **Within-group repeated measures ANOVA (MSPSS-family,** friend, significant others)

```
GLM T0_MSPSS_fam T1_MSPSS_fam T2_MSPSS_fam T0_MSPSS_frd
T1_MSPSS_frd T2_MSPSS_frd T0_MSPSS_sig
    T1_MSPSS_sig T2_MSPSS_sig BY Group
  /WSFACTOR=time 3 Polynomial
  /MEASURE=MSPSS_family MSPSS_friend MSPSS_sig
  /METHOD=SSTYPE(3)
  /PLOT=PROFILE(time*Group) TYPE=LINE ERRORBAR=CI
MEANREFERENCE=NO YAXIS=AUTO
  /EMMEANS=TABLES(OVERALL)
  /EMMEANS=TABLES(time) COMPARE ADJ(BONFERRONI)
  /EMMEANS=TABLES(Group) COMPARE ADJ(BONFERRONI)
  /EMMEANS=TABLES(Group*time)
  /CRITERIA=ALPHA(.05)
  /WSDESIGN=time
  /DESIGN=Group.
```

13. Between-group ANOVA and age*group interaction (PHQ9)

```
UNIANOVA T1_PHQ9_Total BY Group Age_binary Sex
Education_binary Marital_binary Religion_binary
    Employment Treatment
  /METHOD=SSTYPE(3)
  /INTERCEPT=INCLUDE
  /PLOT=PROFILE(Group*Age_binary Age_binary*Group) TYPE=LINE
ERRORBAR=NO MEANREFERENCE=NO YAXIS=AUTO
  /EMMEANS=TABLES(OVERALL)
  /EMMEANS=TABLES(Group) COMPARE ADJ(BONFERRONI)
  /EMMEANS=TABLES(Age_binary) COMPARE ADJ(BONFERRONI)
  /EMMEANS=TABLES(Sex) COMPARE ADJ(BONFERRONI)
  /EMMEANS=TABLES(Education_binary) COMPARE ADJ(BONFERRONI)
  /EMMEANS=TABLES(Marital_binary) COMPARE ADJ(BONFERRONI)
  /EMMEANS=TABLES(Religion_binary) COMPARE ADJ(BONFERRONI)
```

```
  /EMMEANS=TABLES(Employment) COMPARE ADJ(BONFERRONI)
  /EMMEANS=TABLES(Treatment) COMPARE ADJ(BONFERRONI)
  /EMMEANS=TABLES(Age_binary*Group)
  /PRINT ETASQ DESCRIPTIVE PARAMETER HOMOGENEITY OPOWER
  /CRITERIA=ALPHA(.05)
  /DESIGN=Group Age_binary Sex Education_binary Marital_binary
Religion_binary Employment Treatment
    Age_binary*Group.
```

14. Between-group ANOVA and age*group interaction (GAD7)

```
UNIANOVA T1_GAD7_Total BY Group Age_binary Sex
Education_binary Marital_binary Religion_binary
    Employment Treatment
  /METHOD=SSTYPE(3)
  /INTERCEPT=INCLUDE
  /PLOT=PROFILE(Group*Age_binary Age_binary*Group) TYPE=LINE
ERRORBAR=NO MEANREFERENCE=NO YAXIS=AUTO
  /EMMEANS=TABLES(OVERALL)
  /EMMEANS=TABLES(Group) COMPARE ADJ(BONFERRONI)
  /EMMEANS=TABLES(Age_binary) COMPARE ADJ(BONFERRONI)
  /EMMEANS=TABLES(Sex) COMPARE ADJ(BONFERRONI)
  /EMMEANS=TABLES(Education_binary) COMPARE ADJ(BONFERRONI)
  /EMMEANS=TABLES(Marital_binary) COMPARE ADJ(BONFERRONI)
  /EMMEANS=TABLES(Religion_binary) COMPARE ADJ(BONFERRONI)
  /EMMEANS=TABLES(Employment) COMPARE ADJ(BONFERRONI)
  /EMMEANS=TABLES(Treatment) COMPARE ADJ(BONFERRONI)
  /EMMEANS=TABLES(Age_binary*Group)
  /PRINT ETASQ DESCRIPTIVE PARAMETER HOMOGENEITY OPOWER
  /CRITERIA=ALPHA(.05)
  /DESIGN=Group Age_binary Sex Education_binary Marital_binary
Religion_binary Employment Treatment
    Age_binary*Group.
```

15. Between-group ANOVA and age*group interaction (DSES)

```
UNIANOVA T1_DSES_Total BY Group Age_binary Sex
Education_binary Marital_binary Religion_binary
    Employment Treatment
  /METHOD=SSTYPE(3)
  /INTERCEPT=INCLUDE
  /PLOT=PROFILE(Group*Age_binary Age_binary*Group) TYPE=LINE
ERRORBAR=NO MEANREFERENCE=NO YAXIS=AUTO
  /EMMEANS=TABLES(OVERALL)
  /EMMEANS=TABLES(Group) COMPARE ADJ(BONFERRONI)
  /EMMEANS=TABLES(Age_binary) COMPARE ADJ(BONFERRONI)
  /EMMEANS=TABLES(Sex) COMPARE ADJ(BONFERRONI)
  /EMMEANS=TABLES(Education_binary) COMPARE ADJ(BONFERRONI)
  /EMMEANS=TABLES(Marital_binary) COMPARE ADJ(BONFERRONI)
  /EMMEANS=TABLES(Religion_binary) COMPARE ADJ(BONFERRONI)
  /EMMEANS=TABLES(Employment) COMPARE ADJ(BONFERRONI)
```

```
  /EMMEANS=TABLES(Treatment) COMPARE ADJ(BONFERRONI)
  /EMMEANS=TABLES(Age_binary*Group)
  /PRINT ETASQ DESCRIPTIVE PARAMETER HOMOGENEITY OPOWER
  /CRITERIA=ALPHA(.05)
  /DESIGN=Group Age_binary Sex Education_binary Marital_binary
Religion_binary Employment Treatment
    Age_binary*Group.
```

16. Between-group ANOVA and age*group interaction (SHS)

```
UNIANOVA T1_SHS_Total BY Group Age_binary Sex Education_binary
Marital_binary Religion_binary
    Employment Treatment
  /METHOD=SSTYPE(3)
  /INTERCEPT=INCLUDE
  /PLOT=PROFILE(Group*Age_binary Age_binary*Group) TYPE=LINE
ERRORBAR=NO MEANREFERENCE=NO YAXIS=AUTO
  /EMMEANS=TABLES(OVERALL)
  /EMMEANS=TABLES(Group) COMPARE ADJ(BONFERRONI)
  /EMMEANS=TABLES(Age_binary) COMPARE ADJ(BONFERRONI)
  /EMMEANS=TABLES(Sex) COMPARE ADJ(BONFERRONI)
  /EMMEANS=TABLES(Education_binary) COMPARE ADJ(BONFERRONI)
  /EMMEANS=TABLES(Marital_binary) COMPARE ADJ(BONFERRONI)
  /EMMEANS=TABLES(Religion_binary) COMPARE ADJ(BONFERRONI)
  /EMMEANS=TABLES(Employment) COMPARE ADJ(BONFERRONI)
  /EMMEANS=TABLES(Treatment) COMPARE ADJ(BONFERRONI)
  /EMMEANS=TABLES(Age_binary*Group)
  /PRINT ETASQ DESCRIPTIVE PARAMETER HOMOGENEITY OPOWER
  /CRITERIA=ALPHA(.05)
  /DESIGN=Group Age_binary Sex Education_binary Marital_binary
Religion_binary Employment Treatment
    Age_binary*Group.
```

17. **Between-group ANOVA and age*group interaction (SHS-agency)**

```
UNIANOVA T1_SHS_Agency BY Group Age_binary Sex
Education_binary Marital_binary Religion_binary
    Employment Treatment
  /METHOD=SSTYPE(3)
  /INTERCEPT=INCLUDE
  /PLOT=PROFILE(Group*Age_binary Age_binary*Group) TYPE=LINE
ERRORBAR=NO MEANREFERENCE=NO YAXIS=AUTO
  /EMMEANS=TABLES(OVERALL)
  /EMMEANS=TABLES(Group) COMPARE ADJ(BONFERRONI)
  /EMMEANS=TABLES(Age_binary) COMPARE ADJ(BONFERRONI)
  /EMMEANS=TABLES(Sex) COMPARE ADJ(BONFERRONI)
  /EMMEANS=TABLES(Education_binary) COMPARE ADJ(BONFERRONI)
  /EMMEANS=TABLES(Marital_binary) COMPARE ADJ(BONFERRONI)
  /EMMEANS=TABLES(Religion_binary) COMPARE ADJ(BONFERRONI)
  /EMMEANS=TABLES(Employment) COMPARE ADJ(BONFERRONI)
  /EMMEANS=TABLES(Treatment) COMPARE ADJ(BONFERRONI)
```

```
  /EMMEANS=TABLES(Age_binary*Group)
  /PRINT ETASQ DESCRIPTIVE PARAMETER HOMOGENEITY OPOWER
  /CRITERIA=ALPHA(.05)
  /DESIGN=Group Age_binary Sex Education_binary Marital_binary
Religion_binary Employment Treatment
    Age_binary*Group.
```

18. Between-group ANOVA and age*group interaction (SHS-pathway)

```
UNIANOVA T1_SHS_Pathway BY Group Age_binary Sex
Education_binary Marital_binary Religion_binary
    Employment Treatment
  /METHOD=SSTYPE(3)
  /INTERCEPT=INCLUDE
  /PLOT=PROFILE(Group*Age_binary Age_binary*Group) TYPE=LINE
ERRORBAR=NO MEANREFERENCE=NO YAXIS=AUTO
  /EMMEANS=TABLES(OVERALL)
  /EMMEANS=TABLES(Group) COMPARE ADJ(BONFERRONI)
  /EMMEANS=TABLES(Age_binary) COMPARE ADJ(BONFERRONI)
  /EMMEANS=TABLES(Sex) COMPARE ADJ(BONFERRONI)
  /EMMEANS=TABLES(Education_binary) COMPARE ADJ(BONFERRONI)
  /EMMEANS=TABLES(Marital_binary) COMPARE ADJ(BONFERRONI)
  /EMMEANS=TABLES(Religion_binary) COMPARE ADJ(BONFERRONI)
  /EMMEANS=TABLES(Employment) COMPARE ADJ(BONFERRONI)
  /EMMEANS=TABLES(Treatment) COMPARE ADJ(BONFERRONI)
  /EMMEANS=TABLES(Age_binary*Group)
  /PRINT ETASQ DESCRIPTIVE PARAMETER HOMOGENEITY OPOWER
  /CRITERIA=ALPHA(.05)
  /DESIGN=Group Age_binary Sex Education_binary Marital_binary
Religion_binary Employment Treatment
    Age_binary*Group.
```

19. Between-group ANOVA and age*group interaction (MLQ)

```
UNIANOVA T1_MLQ_Total BY Group Age_binary Sex Education_binary
Marital_binary Religion_binary
    Employment Treatment
  /METHOD=SSTYPE(3)
  /INTERCEPT=INCLUDE
  /PLOT=PROFILE(Group*Age_binary Age_binary*Group) TYPE=LINE
ERRORBAR=NO MEANREFERENCE=NO YAXIS=AUTO
  /EMMEANS=TABLES(OVERALL)
  /EMMEANS=TABLES(Group) COMPARE ADJ(BONFERRONI)
  /EMMEANS=TABLES(Age_binary) COMPARE ADJ(BONFERRONI)
  /EMMEANS=TABLES(Sex) COMPARE ADJ(BONFERRONI)
  /EMMEANS=TABLES(Education_binary) COMPARE ADJ(BONFERRONI)
  /EMMEANS=TABLES(Marital_binary) COMPARE ADJ(BONFERRONI)
  /EMMEANS=TABLES(Religion_binary) COMPARE ADJ(BONFERRONI)
  /EMMEANS=TABLES(Employment) COMPARE ADJ(BONFERRONI)
  /EMMEANS=TABLES(Treatment) COMPARE ADJ(BONFERRONI)
  /EMMEANS=TABLES(Age_binary*Group)
```

```
  /PRINT ETASQ DESCRIPTIVE PARAMETER HOMOGENEITY OPOWER
  /CRITERIA=ALPHA(.05)
  /DESIGN=Group Age_binary Sex Education_binary Marital_binary
Religion_binary Employment Treatment
    Age_binary*Group.
```

20. Between-group ANOVA and age*group interaction (MLQ-presence)

```
UNIANOVA T1_MLQ_Presence BY Group Age_binary Sex
Education_binary Marital_binary Religion_binary
    Employment Treatment
  /METHOD=SSTYPE(3)
  /INTERCEPT=INCLUDE
  /PLOT=PROFILE(Group*Age_binary Age_binary*Group) TYPE=LINE
ERRORBAR=NO MEANREFERENCE=NO YAXIS=AUTO
  /EMMEANS=TABLES(OVERALL)
  /EMMEANS=TABLES(Group) COMPARE ADJ(BONFERRONI)
  /EMMEANS=TABLES(Age_binary) COMPARE ADJ(BONFERRONI)
  /EMMEANS=TABLES(Sex) COMPARE ADJ(BONFERRONI)
  /EMMEANS=TABLES(Education_binary) COMPARE ADJ(BONFERRONI)
  /EMMEANS=TABLES(Marital_binary) COMPARE ADJ(BONFERRONI)
  /EMMEANS=TABLES(Religion_binary) COMPARE ADJ(BONFERRONI)
  /EMMEANS=TABLES(Employment) COMPARE ADJ(BONFERRONI)
  /EMMEANS=TABLES(Treatment) COMPARE ADJ(BONFERRONI)
  /EMMEANS=TABLES(Age_binary*Group)
  /PRINT ETASQ DESCRIPTIVE PARAMETER HOMOGENEITY OPOWER
  /CRITERIA=ALPHA(.05)
  /DESIGN=Group Age_binary Sex Education_binary Marital_binary
Religion_binary Employment Treatment
    Age_binary*Group.
```

21. Between-group ANOVA and age*group interaction (MLQ-search)

```
UNIANOVA T1_MLQ_Search BY Group Age_binary Sex
Education_binary Marital_binary Religion_binary
    Employment Treatment
  /METHOD=SSTYPE(3)
  /INTERCEPT=INCLUDE
  /PLOT=PROFILE(Group*Age_binary Age_binary*Group) TYPE=LINE
ERRORBAR=NO MEANREFERENCE=NO YAXIS=AUTO
  /EMMEANS=TABLES(OVERALL)
  /EMMEANS=TABLES(Group) COMPARE ADJ(BONFERRONI)
  /EMMEANS=TABLES(Age_binary) COMPARE ADJ(BONFERRONI)
  /EMMEANS=TABLES(Sex) COMPARE ADJ(BONFERRONI)
  /EMMEANS=TABLES(Education_binary) COMPARE ADJ(BONFERRONI)
  /EMMEANS=TABLES(Marital_binary) COMPARE ADJ(BONFERRONI)
  /EMMEANS=TABLES(Religion_binary) COMPARE ADJ(BONFERRONI)
  /EMMEANS=TABLES(Employment) COMPARE ADJ(BONFERRONI)
  /EMMEANS=TABLES(Treatment) COMPARE ADJ(BONFERRONI)
  /EMMEANS=TABLES(Age_binary*Group)
  /PRINT ETASQ DESCRIPTIVE PARAMETER HOMOGENEITY OPOWER
```

```
  /CRITERIA=ALPHA(.05)
  /DESIGN=Group Age_binary Sex Education_binary Marital_binary
Religion_binary Employment Treatment
    Age_binary*Group.
```

22. Between-group ANOVA and age*group interaction (RSES)

```
UNIANOVA T1_RSES_Total BY Group Age_binary Sex
Education_binary Marital_binary Religion_binary
    Employment Treatment
  /METHOD=SSTYPE(3)
  /INTERCEPT=INCLUDE
  /PLOT=PROFILE(Group*Age_binary Age_binary*Group) TYPE=LINE
ERRORBAR=NO MEANREFERENCE=NO YAXIS=AUTO
  /EMMEANS=TABLES(OVERALL)
  /EMMEANS=TABLES(Group) COMPARE ADJ(BONFERRONI)
  /EMMEANS=TABLES(Age_binary) COMPARE ADJ(BONFERRONI)
  /EMMEANS=TABLES(Sex) COMPARE ADJ(BONFERRONI)
  /EMMEANS=TABLES(Education_binary) COMPARE ADJ(BONFERRONI)
  /EMMEANS=TABLES(Marital_binary) COMPARE ADJ(BONFERRONI)
  /EMMEANS=TABLES(Religion_binary) COMPARE ADJ(BONFERRONI)
  /EMMEANS=TABLES(Employment) COMPARE ADJ(BONFERRONI)
  /EMMEANS=TABLES(Treatment) COMPARE ADJ(BONFERRONI)
  /EMMEANS=TABLES(Age_binary*Group)
  /PRINT ETASQ DESCRIPTIVE PARAMETER HOMOGENEITY OPOWER
  /CRITERIA=ALPHA(.05)
  /DESIGN=Group Age_binary Sex Education_binary Marital_binary
Religion_binary Employment Treatment
    Age_binary*Group.
```

23. Between-group ANOVA and age*group interaction (MSPSS)

```
UNIANOVA T1_MSPSS_Total BY Group Age_binary Sex
Education_binary Marital_binary Religion_binary
    Employment Treatment
  /METHOD=SSTYPE(3)
  /INTERCEPT=INCLUDE
  /PLOT=PROFILE(Group*Age_binary Age_binary*Group) TYPE=LINE
ERRORBAR=NO MEANREFERENCE=NO YAXIS=AUTO
  /EMMEANS=TABLES(OVERALL)
  /EMMEANS=TABLES(Group) COMPARE ADJ(BONFERRONI)
  /EMMEANS=TABLES(Age_binary) COMPARE ADJ(BONFERRONI)
  /EMMEANS=TABLES(Sex) COMPARE ADJ(BONFERRONI)
  /EMMEANS=TABLES(Education_binary) COMPARE ADJ(BONFERRONI)
  /EMMEANS=TABLES(Marital_binary) COMPARE ADJ(BONFERRONI)
  /EMMEANS=TABLES(Religion_binary) COMPARE ADJ(BONFERRONI)
  /EMMEANS=TABLES(Employment) COMPARE ADJ(BONFERRONI)
  /EMMEANS=TABLES(Treatment) COMPARE ADJ(BONFERRONI)
  /EMMEANS=TABLES(Age_binary*Group)
  /PRINT ETASQ DESCRIPTIVE PARAMETER HOMOGENEITY OPOWER
  /CRITERIA=ALPHA(.05)
```

```
  /DESIGN=Group Age_binary Sex Education_binary Marital_binary
Religion_binary Employment Treatment
    Age_binary*Group.
```

24. Between-group ANOVA and age*group interaction (MSPSS-family)

```
UNIANOVA T1_MSPSS_fam BY Group Age_binary Sex Education_binary
Marital_binary Religion_binary
    Employment Treatment
  /METHOD=SSTYPE(3)
  /INTERCEPT=INCLUDE
  /PLOT=PROFILE(Group*Age_binary Age_binary*Group) TYPE=LINE
ERRORBAR=NO MEANREFERENCE=NO YAXIS=AUTO
  /EMMEANS=TABLES(OVERALL)
  /EMMEANS=TABLES(Group) COMPARE ADJ(BONFERRONI)
  /EMMEANS=TABLES(Age_binary) COMPARE ADJ(BONFERRONI)
  /EMMEANS=TABLES(Sex) COMPARE ADJ(BONFERRONI)
  /EMMEANS=TABLES(Education_binary) COMPARE ADJ(BONFERRONI)
  /EMMEANS=TABLES(Marital_binary) COMPARE ADJ(BONFERRONI)
  /EMMEANS=TABLES(Religion_binary) COMPARE ADJ(BONFERRONI)
  /EMMEANS=TABLES(Employment) COMPARE ADJ(BONFERRONI)
  /EMMEANS=TABLES(Treatment) COMPARE ADJ(BONFERRONI)
  /EMMEANS=TABLES(Age_binary*Group)
  /PRINT ETASQ DESCRIPTIVE PARAMETER HOMOGENEITY OPOWER
  /CRITERIA=ALPHA(.05)
  /DESIGN=Group Age_binary Sex Education_binary Marital_binary
Religion_binary Employment Treatment
    Age_binary*Group.
```

25. Between-group ANOVA and age*group interaction (MSPSS-friend)

```
UNIANOVA T1_MSPSS_frd BY Group Age_binary Sex Education_binary
Marital_binary Religion_binary
    Employment Treatment
  /METHOD=SSTYPE(3)
  /INTERCEPT=INCLUDE
  /PLOT=PROFILE(Group*Age_binary Age_binary*Group) TYPE=LINE
ERRORBAR=NO MEANREFERENCE=NO YAXIS=AUTO
  /EMMEANS=TABLES(OVERALL)
  /EMMEANS=TABLES(Group) COMPARE ADJ(BONFERRONI)
  /EMMEANS=TABLES(Age_binary) COMPARE ADJ(BONFERRONI)
  /EMMEANS=TABLES(Sex) COMPARE ADJ(BONFERRONI)
  /EMMEANS=TABLES(Education_binary) COMPARE ADJ(BONFERRONI)
  /EMMEANS=TABLES(Marital_binary) COMPARE ADJ(BONFERRONI)
  /EMMEANS=TABLES(Religion_binary) COMPARE ADJ(BONFERRONI)
  /EMMEANS=TABLES(Employment) COMPARE ADJ(BONFERRONI)
  /EMMEANS=TABLES(Treatment) COMPARE ADJ(BONFERRONI)
  /EMMEANS=TABLES(Age_binary*Group)
  /PRINT ETASQ DESCRIPTIVE PARAMETER HOMOGENEITY OPOWER
  /CRITERIA=ALPHA(.05)
```

```
  /DESIGN=Group Age_binary Sex Education_binary Marital_binary
Religion_binary Employment Treatment
    Age_binary*Group.
```

26. Between-group ANOVA and age*group interaction (MSPSS-significant others)

```
UNIANOVA T1_MSPSS_sig BY Group Age_binary Sex Education_binary
Marital_binary Religion_binary
    Employment Treatment
  /METHOD=SSTYPE(3)
  /INTERCEPT=INCLUDE
  /PLOT=PROFILE(Group*Age_binary Age_binary*Group) TYPE=LINE
ERRORBAR=NO MEANREFERENCE=NO YAXIS=AUTO
  /EMMEANS=TABLES(OVERALL)
  /EMMEANS=TABLES(Group) COMPARE ADJ(BONFERRONI)
  /EMMEANS=TABLES(Age_binary) COMPARE ADJ(BONFERRONI)
  /EMMEANS=TABLES(Sex) COMPARE ADJ(BONFERRONI)
  /EMMEANS=TABLES(Education_binary) COMPARE ADJ(BONFERRONI)
  /EMMEANS=TABLES(Marital_binary) COMPARE ADJ(BONFERRONI)
  /EMMEANS=TABLES(Religion_binary) COMPARE ADJ(BONFERRONI)
  /EMMEANS=TABLES(Employment) COMPARE ADJ(BONFERRONI)
  /EMMEANS=TABLES(Treatment) COMPARE ADJ(BONFERRONI)
  /EMMEANS=TABLES(Age_binary*Group)
  /PRINT ETASQ DESCRIPTIVE PARAMETER HOMOGENEITY OPOWER
  /CRITERIA=ALPHA(.05)
  /DESIGN=Group Age_binary Sex Education_binary Marital_binary
Religion_binary Employment Treatment
    Age_binary*Group.
```
